# Periaortic inflammation after endoleak repair: a risk to watch

**DOI:** 10.1186/s42155-026-00718-3

**Published:** 2026-06-27

**Authors:** Marilia Bernadette Voigt, Alexander Böhner, Mohammed Bahaaeldin, Julia Wagenpfeil, Tatjana Dell, Alexander Isaak, Claus-Christian Pieper, Julian A. Luetkens, Patrick A. Kupczyk, Carsten Meyer, Alexander Kania, Daniel Kuetting

**Affiliations:** 1 Department of Diagnostic and Interventional Radiology, University Hospital Bonn, Bonn, Germany; 2 Department of Visceral and Vascular Surgery, University Hospital Bonn, Bonn, Germany

**Keywords:** Endovascular aortic repair, Endoleak repair, Endoleak embolization, Periaortic inflammation, Peri-aortitis, NCBA, EVOH

## Abstract

**Purpose:**

To evaluate the occurrence of periaortic inflammation, including abscess formation, as a complication following endoleak repair after endovascular aortic repair.

**Materials and methods:**

This retrospective single-center study analyzed 88 EVAR revisions for endoleak treatment performed in 59 patients between 2015 and 2025. Patients underwent one to three revision procedures. Endoleaks were classified by type, and treatment was performed via transarterial access or direct sac puncture with a posterior/translumbar access route. Embolization materials for type II endoleaks included n-butyl-2-cyanoacrylate, ethylene vinyl alcohol, and coils, while stent-graft extensions and transmural fixation were used for type I and III endoleaks.

**Results:**

Of the 59 patients, 38 (64.4%) underwent one revision, 13 (22.0%) two revisions, and 8 (13.6%) three revisions. Treated endoleaks included type I (19.3%), type II (77.3%), type III (2.3%), and combined type II/III (1.1%). A total of 73 procedures (83.0%) were performed via transarterial access and 15 (17.0%) via direct sac puncture. Follow-up CT identified 6 cases of periaortic inflammation after type II endoleak repair, presenting with soft tissue mantle, fat stranding, or abscess formation. Symptom onset ranged from 33 to 108 days after the most recent intervention. No significant association was found between periaortic inflammation and access route or embolic material.

**Conclusion:**

Periaortic inflammation and abscess formation are possible severe complications after endoleak repair following endovascular aortic repair. To date, these inflammatory changes have not been described systematically in the literature and warrant further investigation.

## Introduction

Endovascular aortic repair (EVAR) has become a widely accepted treatment for abdominal aortic aneurysms (AAA), offering lower perioperative morbidity and mortality compared to open repair. However, post-EVAR imaging can reveal endoleaks (ELs) as technique inherent drawbacks, which can compromise long-term efficacy and necessitate reintervention.

A systematic review from 2018 on the treatment of type II ELs reports low overall complication rates, yet infection or periaortic inflammation are not listed among the potential adverse outcomes [1–4]. Although most post-interventional courses are uneventful, there is a growing recognition of inflammatory complications following embolization procedures. To date, only isolated case reports and small series have described this phenomenon, without systematic analysis of its frequency, characteristics, or clinical implications [5–8].

The aim of this study was to assess the frequency of periaortic inflammation following EL embolization and to explore whether its occurrence is associated with the choice of access technique or the embolic material used.

## Materials and methods

This retrospective, single-center observational study was conducted at a tertiary care university hospital. The institutional review board approved the study protocol and waived the requirement for written informed consent due to the retrospective nature of data collection and analysis (application number 303/16).

### Patient selection

All patients who underwent EVAR followed by secondary intervention for type I, II, or III EL between January 2015 and January 2025 were selected. Patients with open aortic repair, aorto-iliac bypass surgery, or thromboendarterectomy (TEA) were excluded.

### Patient demographics

A total of 59 patients (including 5 women and 54 men) obtained one, two or three EL repair interventions. Mean patient age was 75 years (± 9.1; range from 40 to 89 years). Patient demographics and procedural details were extracted. Each case was reviewed for the type and anatomical location of the EL, as well as the approach to revision therapy – categorized as either direct sac puncture or transarterial technique. Embolic or prosthetic materials used were documented.

### Data collection

Post-interventional clinical follow-up data were assessed for any symptoms suggestive of inflammatory or infectious complications. Laboratory parameters were reviewed. Where available, blood culture results were recorded, including identification of microbial pathogens.

All follow-up CT examinations were analyzed for signs of periaortic or retroperitoneal pathology. FDG-PET imaging, if available, was evaluated for periaortic or retroperitoneal radiotracer uptake. In cases with suspicious or unclear imaging findings, results of image-guided biopsies and corresponding histopathological analysis were included.

Finally, therapeutic consequences of positive findings were recorded.

### Statistical analysis

Descriptive statistics were used to summarize baseline and procedural characteristics. Categorical variables are presented as absolute and relative frequencies, while continuous variables are expressed as mean ± standard deviation (SD) for normally distributed data or as median with interquartile range (IQR) for non-normally distributed data.

Normality of continuous variables was assessed using the Shapiro–Wilk test. For group comparisons, the independent samples t-test was used for normally distributed continuous variables, and the Mann–Whitney U test for non-normally distributed variables. Categorical data were compared using Fisher’s exact test due to small sample sizes and binary outcomes.

A two-tailed *p*-value < 0.05 was considered statistically significant.

## Results

### Endoleak revision

A total of 59 patients underwent 88 EL reinterventions following prior EVAR over the 10-year study period. Of these, 38 patients (64.4%) underwent one reintervention, 12 patients (22.0%) required two, and 8 patients (13.6%) underwent three reinterventions. An overview of the patients’ comorbidities can be found in Table 1.

**Table 1:** Patient characteristics

Patient characteristics (*n* = 59)	
Mean age in years	74 (40—89)
Gender	
Female	5 (8.5%)
Male	54 (91.5%)
Smoking	10 (32.3%)
Overweight	14 (45.2%)
Hypertension	19 (61.3%)
Dyslipidemia	16 (51.6%)
Peripheral artery disease	9 (29.0%)
Abdominal aortic aneurysm	6 (19.4%)
Type II diabetes	6 (19.4%)

Among all 88 procedures, EL type II was the most common indication, observed in 68 cases (77.3%), followed by type I in 17 cases (19.3%) and type III in 2 cases (2.3%). One patient (1.1%) presented with a combined type II and type III EL.

Puncture sites were disinfected with povidone-iodine. Access was transarterial/transfemoral in 73 interventions (83.0%), while percutaneous direct sac puncture with a posterior/translumbar access route of the EL was performed in 15 cases (17.0%). Type II EL involved the following vessels: lumbar arteries (*n* = 43), inferior mesenteric artery (IMA) (*n* = 16), median sacral artery (*n* = 3) and internal iliac arteries (*n* = 3). In 6 cases, the origin vessel remained unknown.

**Fig. 1:**
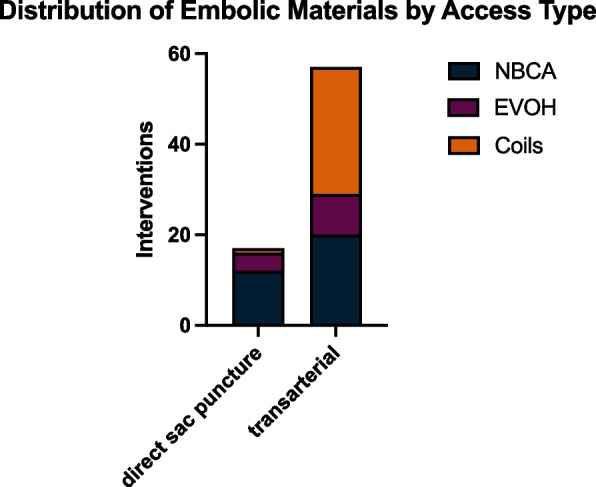
Embolic agents used in endovascular vs. direct puncture approaches. Distribution of embolic materials used for the treatment of type II endoleaks, stratified by access type (endovascular vs. direct puncture). Note that multiple materials could be used in a single procedure

Embolization materials or devices used included NBCA (*n* = 32), EVOH (*n* = 13), coils (*n* = 29), stent extensions (*n* = 19), and transmural fixation (*n* = 1). In 6 cases, more than one material was used within a single procedure. The distribution of embolic agents and adjunctive devices across EL types is summarized in Table 2 and across the different access route in Fig. 1.

**Table 2:** Distribution of embolic agents and adjunctive devices across endoleak types

Embolic agent or adjunctive device	EL Type I (*n* = 17)	EL Type II (*n* = 68)	EL Type III (*n* = 2)	EL Type II and III combined (*n *= 1)	Total Interventions (*n* = 88)
NBCA	0	32	0	0	32
EVOH	0	13	0	0	13
Coils	0	28	0	1	29
Stent Graft Extension	16	0	2	1	19
Transmural Fixation	1	0	0	0	1

Note that multiple materials were used in six procedures

The use of NBCA was more common in the percutaneous group (80.0% vs. 37.0%, *p* = 0.004), while coil embolization was more common in the endovascular group (51.6% vs. 6.7%, *p* = 0.002). EVOH (*p* = 0.458) was used infrequently and without significant group difference.

### Periaortic inflammation

In 6 cases (6.8%) patients developed periaortic inflammation, all of whom had undergone treatment for type II ELs. Within type II EL interventions, periaortic inflammation was observed in 7.4% of endovascular cases and in 13.3% of percutaneous cases, indicating no statistically significant difference (*p* = 0.604).

Among the 6 patients who developed periaortic inflammation, embolization materials used prior to symptom onset were NBCA alone (*n* = 4), EVOH (*n* = 2) and coils (*n* = 2). In two cases a combination therapy of NBCA/EVOH and NBCA/coils was applied. There was no statistically significant difference in the use of NBCA, EVOH-based embolic agents or coils between patients with and without periaortic inflammation (*p* = 0.673, *p* = 0.315 and *p* = 0.671, respectively). Two of the six patients with periaortic inflammation had chronic kidney disease, while no other relevant predisposing risk factors for infection, such as diabetes mellitus or immunosuppression, were present.

**Fig. 2:**
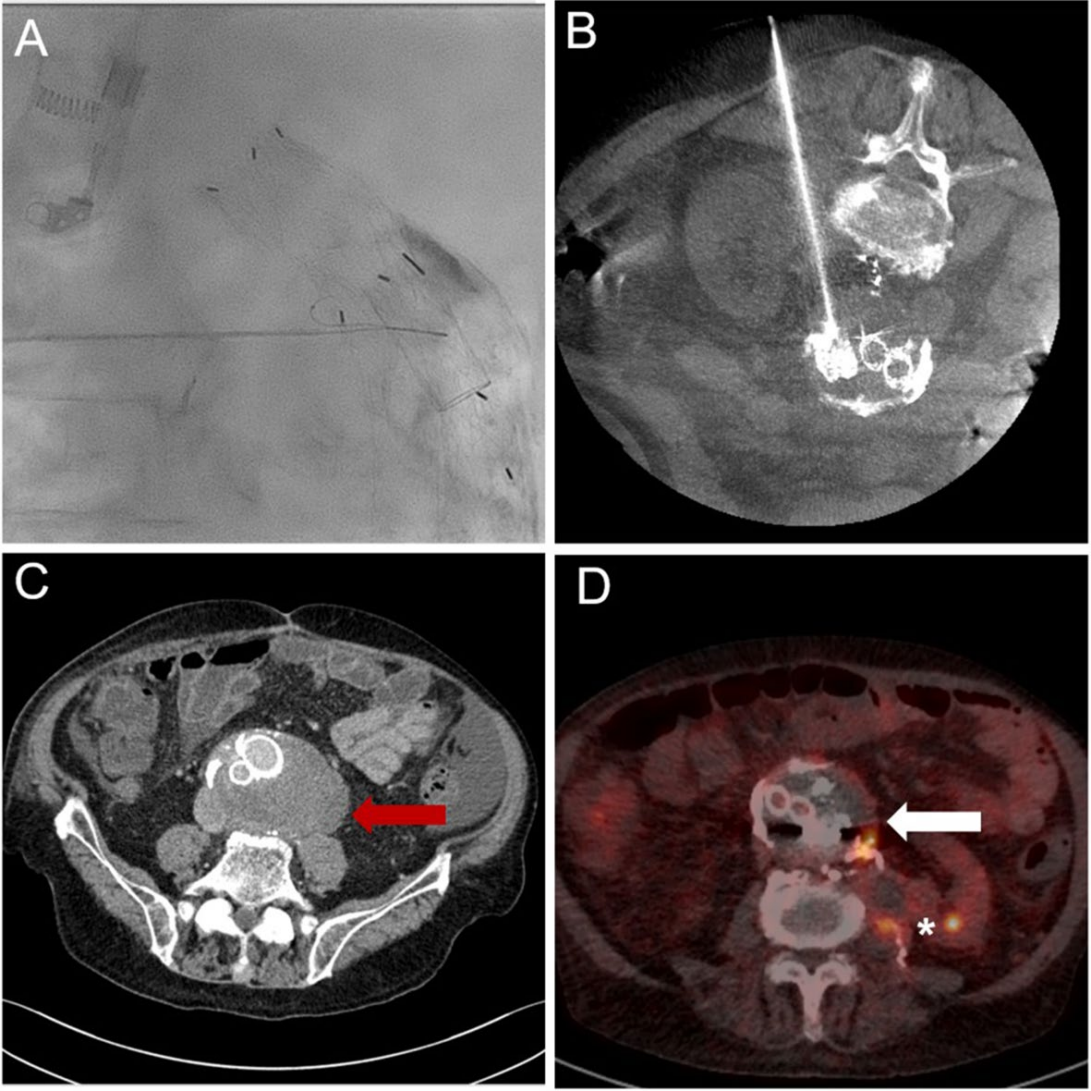
Representative case of a 78-year-old male patient undergoing a second attempt of type II endoleak embolization via direct sac puncture from the third right lumbar artery using EVOH. **A**, **B** Intra-procedural images of the embolization. **C** Follow-up CT 33 days after the procedure showing a circumferential periaortic soft-tissue mantle in the presence of clinical symptoms and elevated inflammatory markers. **D** FDG-PET/CT performed 11 days later demonstrating increased tracer uptake along the aortic wall (white arrow) and ring-shaped uptake within the left psoas muscle (white asterisk), consistent with abscess formation subsequently confirmed by CT

Two patients became symptomatic following percutaneous direct sac puncture (after 33 and 106 days), while four patients developed symptoms after transfemoral endovascular procedures (after 62, 63, 108 and 109 days, respectively), with a median of 85 days and an IQR of 45 days. Clinical presentations included systemic signs (e.g., fever, malaise, abdominal pain) and were accompanied by elevated inflammatory markers. All 6 patients showed positive imaging findings on post-interventional CT, with varying combinations of soft-tissue stranding, periaortic fluid, and fat stranding. FDG-PET/CT, available in four patients, confirmed increased uptake at the inflammatory sites. Inflammatory markers (CRP, PCT, leukocyte count) were elevated in all patients with periaortic inflammation, while no abnormal laboratory parameters were observed in patients without inflammation. A representative case is shown in Fig. 2.

Blood cultures obtained in all 6 patients with CT morphologic periaortic inflammation were positive in 4 cases, revealing organisms such as gram-positive cocci, Escherichia coli (E. coli), Pseudomonas aeruginosa (P. aeruginosa) and Cutibacterium acnes (C. acnes). Histopathological analysis was obtained in three patients and revealed xanthogranulomatous and chronic active inflammation in all three biopsy samples, with evidence of necrosis, vascular wall involvement, and mixed-cell infiltrates. All patients required active treatment including intravenous antibiotics, drainage, or surgical conversion to open repair. Three patients fully recovered after prolonged hospitalization including intensive care, two patients showed persistent CT findings over the course of more than two years – one of whom remains on long-term antibiotic therapy to date – and one patient died of hemorrhagic shock and sepsis following open conversion.

A detailed overview of the clinical course, laboratory and imaging findings, as well as the materials and access routes used in the 6 patients with periaortic inflammation is provided in Table 3.

**Table 3:** Clinical and procedural characteristics of patients with post-interventional periaortic inflammation

Patient	Revision attempt	Access	Embolization material	Onset (d)	Symptoms	Elevated inflammatory markers	Blood cultures	CT	FDG-PET/CT	Histopathological report	Therapy	Outcome
1	first (1/1)	transfemoral	coils	109	fever, abdominal pain, GM	CRP, PCT, leukocyte count	positive (gram-positive cocci)	aortoduodenal fistula with empyema within aortic sack, psoas abscess	N/A	Fibrous thickening of the adventitia with marked xanthogranulomatous inflammation rich in foamy macrophages, consistent with a chronic inflammatory process exhibiting focal acute activity	conversion to open repair with partial aortic resection and vein transplant, psoas drainage, lavage	death on day 14 post operation due to hemorrhagic shock and sepsis
2	first (1/1)	transfemoral	NBCA/coils	63	fever, back pain, GM	CRP	negative	CE periaortic soft tissue mantle with infiltration of left psoas muscle and urether	positive	Chronic active inflammation of the soft tissue characterized by a mixed inflammatory infiltrate composed of lymphocytes, plasma cells, and neutrophilic granulocytes	antibiotics (ciprofloxacin)	persistent findings over the course of 26 months
3	second (2/2)	direct sac puncture	NBCA	106	GM	CRP, PCT	positive (E. coli)	CE periaortic soft tissue mantle, sigmoid fistula	positive	N/A	antibiotics (pip/taz, mero, vanco, unacid)	persistent findings over the course of 24 months, life-long need for antibiotics
4	second (2/2)	direct sac puncture	NBCA/EVOH	33	fever, chills, abdominal pain, GM	CRP, PCT, leukocyte count	positive (gram-positive cocci)	CE periaortic soft tissue mantle, abscess, enlarged left psoas muscle	positive	N/A	antibiotics (piperacillin/tazobac), drainage	reconvalescence
5	second (2/2)	transfemoral	EVOH	108	GM	CRP, PCT	negative	CE periaortic soft tissue mantle, abscess	N/A	Scarred and ossified fat necrosis associated with florid fibrinous inflammation involving necrotic segments of arterial vessel walls	antibiotics, drainage, conversion to laparotomy with explantation of EVAR graft and aortobiiliacal open repair	reconvalescence
6	third (3/3)	transfemoral	NBCA	62	fever, back pain	CRP, PCT, leukocyte count	positive (Pseudomonas aeruginosa and Staph. Lugdonensis)	CE periaortic soft tissue mantle, abscess	positive	N/A	antibiotics, drainage, explantation of EVAR graft and axillobifermoral bypass	??

Summary of clinical symptoms, time to symptom onset after the last endoleak embolization procedure, type of access, embolic materials used, laboratory findings, blood culture results, and imaging findings in the five patients diagnosed with periaortic inflammation following type II endoleak treatment

CT findings refer to the presence of periaortic soft tissue changes, fat stranding, or fluid collections. Positive blood cultures indicate microbiologically confirmed bacteremia. *Abbreviations*: *CABG* coronary artery bypass graft, *CAD* coronary artery disease, *CE* contrast-enhancing, *CRP* C-reactive protein, *EVOH* ethylene vinyl alcohol, *GM* generalized malaise, *NBCA* N-buty cyanoacrylate, *lPCT* procalcitonin

## Discussion

In this retrospective study we systematically assessed periaortic inflammatory changes after EL repair following EVAR. Periaortic inflammation was identified in 6.8% of patients undergoing EL repair after EVAR. All cases occurred after coil and/or fluid embolization, while no inflammatory reactions were observed in patients treated with stent graft implantation or transmural fixation.

Importantly, periaortic inflammation occurred following both transfemoral and posterior direct sac puncture approaches.

Previous reports on periaortic inflammation after EVAR are limited to isolated case reports and small series. In a 2016 case report, a 66-year-old male patient was described, who developed a stent graft infection with C. acnes, an anaerobic gram-positive skin commensal, 9 days after type II EL repair with coil embolization of the IMA via a transfemoral approach [5]. At presentation with fever and abdominal pain, CT imaging revealed a periaortic abscess, necessitating explantation of the stent graft and conversion to open repair.

A 2024 case report describes a 71-year-old male patient, who developed periaortic fat stranding 2 days after transcaval EL embolization with NBCA, which was FDG-positive on PET imaging [6]. Furthermore, CT-guided puncture of the inflamed area revealed Staph. epidermidis and C. acnes. In 2024, another case was reported involving a 75-year-old male patient who presented with fever and lower back pain 5 days after direct sac puncture of a lumbar type II EL and embolization with NBCA. In this case as well, infection of the periaortic tissue with C. acnes was confirmed. The stent graft was explanted and replaced with an aortobiiliac bypass. The patient subsequently died due to an intracranial hemorrhage [7]. In another 2019 case, a patient developed fever two days after direct sac puncture of a type II EL, with subsequent bacteremia due to E. coli and superinfection of the periaortic tissue, including abscess formation with multiple gram-positive gut bacteria [8]. In contrast to these reports, no cases of C. acnes infection and/or other early-onset infectious complications were observed in our study. Overall, C. acnes is considered a rather uncommon pathogen in the context of postoperative infections [9]. Moreover, the onset of symptoms in our series occurred later, typically weeks to months after embolization, suggesting a broader clinical spectrum. The variable time of onset observed suggests that periaortic inflammation may arise from multiple underlying mechanisms. Possible etiologies include sterile inflammatory responses to embolic agents (particularly NBCA or EVOH), necrosis-related inflammation, infection-driven processes, or combinations thereof. This is supported by the fact that only 50% of our patients had positive blood cultures, while histopathological findings in some cases showed features more typical of sterile inflammation, such as xanthogranulomatous reaction or calcified fat necrosis. The persistence of CT abnormalities over more than two years in two patients also argues against a purely infectious etiology. These considerations raise the question whether, in selected cases, adjunctive anti-inflammatory treatment such as corticosteroids (analogous to management strategies in autoimmune aortitis) may complement antibiotic therapy.

Takeuchi et al. recently reported on peri-aortitis in a large EVAR cohort without EL of 1369 patients, identifying an incidence of 0.89% with most cases resolving spontaneously or after antibiotic treatment [10]. 

In contrast, our study focused on periaortic inflammation following EL embolization. We observed a higher incidence of inflammation (6.8%). NBCA can induce a pronounced foreign-body-type inflammatory response with necrotizing vasculitis and subsequenz granulomatous inflammation and fibrosis, while modified cynoacrylate formulations such as GLUBRAN2 exhibit a reduced inflammatory profile due to slower polymerization and lower heat release [11–13]. EVOH-based agents are generally associated with milder chronic inflammatory reactions, although solvent related toxicity, particularly from DMSO, may contribute to vascular and perivascular inflammation [11–13]. In another comparative histopathological analysis of cerebral AVMs, NBCA showed more pronounced vessel wall inflammation, while EVOH-based embolization with Onyx was associated with preserved tissue elasticity [14].

All patients with periaortic inflammation in our study required treatment, including antibiotics, drainage, or conversion to open aortic repair including bypass surgery. 

Our findings demonstrate that periaortic inflammation may occur after EL repair irrespective of the chosen access route, and can result in severe consequences. Given the small number of reports in the literature, it is likely that the true incidence of this complication is underrecognized and underreported. At present, no standardized recommendations exist regarding peri-interventional hygiene management. Based on our experience, we advocate for preventive measures including peri-interventional single-shot antibiotic prophylaxis and thorough surgical cleansing of the puncture site with iodine, rather than simple skin disinfection, regardless of access type. Furthermore, the indication for pre-emptive embolization of arteries such as the IMA or lumbar arteries prior to EVAR to avoid EL should be carefully considered.

Future research should focus on multicenter prospective registries to better define the incidence, risk factors, and natural history of periaortic inflammation after EL repair. Moreover, further study of the immunological and histopathological mechanisms may clarify the pathogenesis and inform targeted therapeutic strategies.

## Conclusion

Periaortic inflammation is an uncommon but clinically relevant complication after EL repair following EVAR. In this study, its occurrence was not associated with the choice of access technique or the embolic material used. Awareness of this entity is essential to avoid misdiagnosis as graft infection and to guide appropriate patient management.

## Data Availability

Not applicable.

## Abbreviations

AAA: Abdominal aortic aneurysm(s)
CRP: C-reactive protein
CT: Computed tomography
DMSO: Dimethyl sulfoxide
EL(s): Endoleak(s)
EVAR: Endovascular aortic repair
EVOH: Ethylene vinyl alcohol
FDG-PET: Fluorodeoxyglucose positron emission tomography
NBCA: N-butyl cyanoacrylate
PCT: Procalcitonin
